# Mild Traumatic Brain Injury-Induced Disruption of the Blood-Brain Barrier Triggers an Atypical Neuronal Response

**DOI:** 10.3389/fncel.2022.821885

**Published:** 2022-02-18

**Authors:** Carmen Munoz-Ballester, Dzenis Mahmutovic, Yusuf Rafiqzad, Alia Korot, Stefanie Robel

**Affiliations:** ^1^Fralin Biomedical Research Institute, Virginia Tech Carilion, Roanoke, VA, United States; ^2^Department of Cell, Developmental and Integrative Biology, University of Alabama at Birmingham, Birmingham, AL, United States; ^3^School of Neuroscience, Virginia Tech Carilion, Blacksburg, VA, United States; ^4^Kenyon College, Gambier, OH, United States

**Keywords:** traumatic brain injury, acute TBI, chronic TBI, spines, primary injury, secondary injury, NeuN, concussion

## Abstract

Mild TBI (mTBI), which affects 75% of TBI survivors or more than 50 million people worldwide each year, can lead to consequences including sleep disturbances, cognitive impairment, mood swings, and post-traumatic epilepsy in a subset of patients. To interrupt the progression of these comorbidities, identifying early pathological events is key. Recent studies have shown that microbleeds, caused by mechanical impact, persist for months after mTBI and are correlated to worse mTBI outcomes. However, the impact of mTBI-induced blood-brain barrier damage on neurons is yet to be revealed. We used a well-characterized mouse model of mTBI that presents with frequent and widespread but size-restricted damage to the blood-brain barrier to assess how neurons respond to exposure of blood-borne factors in this pathological context. We used immunohistochemistry and histology to assess the expression of neuronal proteins in excitatory and inhibitory neurons after mTBI. We observed that the expression of NeuN, Parvalbumin, and CamKII was lost within minutes in areas with blood-brain barrier disruption. Yet, the neurons remained alive and could be detected using a fluorescent Nissl staining even 6 months later. A similar phenotype was observed after exposure of neurons to blood-borne factors due to endothelial cell ablation in the absence of a mechanical impact, suggesting that entrance of blood-borne factors into the brain is sufficient to induce the neuronal atypical response. Changes in postsynaptic spines were observed indicative of functional changes. Thus, this study demonstrates That exposure of neurons to blood-borne factors causes a rapid and sustained loss of neuronal proteins and changes in spine morphology in the absence of neurodegeneration, a finding that is likely relevant to many neuropathologies.

## Introduction

Traumatic brain injury affects more than 69 million people worldwide every year (Dewan et al., 2018), leading to chronic comorbidities such as sleep disorders, neuroendocrine dysregulation, post-traumatic epilepsy, or psychiatric problems (Bramlett and Dietrich, 2015). Some of the candidate mechanisms to induce these comorbidities include edema, focal lesions with tissue loss and subdural hemorrhage (van Asch et al., 2010; Jha et al., 2019; Turtzo et al., 2021). Yet, these mechanisms are not present in mild TBI (mTBI), which affects 75% of TBI survivors. In contrast to moderate and severe TBI with focal injury characterized by primary tissue loss visible upon computed tomography imaging in humans, mTBI presents with diffuse damage due to tissue shearing in the absence of focal injury (Cifu et al., 2009). There is increasing evidence that even a mTBI can lead to severe consequences including sleep disturbances, cognitive impairment, mood swings, and post-traumatic epilepsy (reviewed in Shandra et al., 2019). Thus, to interrupt the progression of these comorbidities, identifying early pathological events is key. Recent studies have shown that microbleeds, which persist for months after TBI, are predictors of worse mTBI outcomes (Griffin et al., 2019). Microbleeds are defined as chronic accumulation of small blood products in the brain tissue due to disruption of the blood-brain barrier (BBB). Yet, the early cellular and molecular events triggered by microbleeds that may be at the root of long-term challenges after mTBI have not been fully identified.

BBB disruption is associated with several neurodegenerative disorders, including multiple sclerosis, Alzheimer’s disease, and early dementia (Petersen et al., 2018). It has been proposed that BBB disruption and the consequent leakage of blood-borne factors induce an increase in the oxidative stress that eventually causes neuronal cell death. Furthermore, many studies have analyzed the impact of blood proteins entering the brain. Fibrinogen (340 kDa) triggers inflammation (Adams et al., 2007), astrocyte boundary formation (Schachtrup et al., 2010), and spine elimination (Merlini et al., 2019). Albumin (66.5 kDa) reduces potassium buffering, favors neuronal hyperexcitability (Ivens et al., 2007), and induces the release of proinflammatory cytokines (Liu et al., 2011). Thrombin (72–263.75 kDa) is associated with cognitive decline (Festoff et al., 2016) and inflammation (Dittmeier et al., 2016). The extent of the vascular and BBB damage determines which factors enter the brain parenchyma. For example, after focal TBI, entire vessels are ruptured, allowing for entrance of all size plasma proteins, immune cells, and erythrocytes.

In a recent study, we assessed the extent and consequences of BBB damage in a mouse model of mTBI, which does not have primary tissue loss and focal lesions. There was frequent and widespread damage to the BBB, characterized by the leakage of the small polar tracer cadaverine (0.9 kDa), which was incorporated by neurons. Yet, the large size plasma protein fibrinogen was deposited rarely. In addition, vessel rupture and immune cell infiltration were sparse suggesting that BBB disruption associated with mTBI is smaller in extent compared to focal TBI (George et al., 2021).

In areas of BBB leakage, instead of the classic astroglial response characterized by astrocyte hypertrophy and the increase in GFAP expression, a subset of astrocytes in the cortical gray matter lost most astrocyte proteins including those involved in brain homeostasis, e.g., Glutamate transporter 1 (Glt1), the potassium channel Kir4.1, glutamine synthetase, connexin43, or S100β. However, these astrocytes remained alive, as demonstrated by the expression of several tdTomato reporters (Shandra et al., 2019). These cells are named “atypical astrocytes” because of the lack of typical reactive astrocyte markers. Exposure of astrocytes to blood plasma proteins in the absence of mechanical injury was sufficient to induce this response (George et al., 2021). Interestingly, microglia activation in areas with BBB damage was mild and transient (Shandra et al., 2019), possibly due to the limited extent of the damage. Yet, how small-size disruptions of the BBB affect neurons, especially early after mTBI, remains unknown.

Here, we assessed the response of neurons to BBB leakage after mTBI. We analyzed the expression of neuronal proteins including NeuN, parvalbumin (PV), and Ca^2+^/calmodulin-dependent protein kinase II (CamKII) in areas of mTBI-induced BBB disruption. We observed that the expression of these proteins was lost. Yet, neurons remained alive and could be detected using a fluorescent Nissl staining. These neurons had less Homer1 postsynaptic puncta, which suggests effects at the functional level in neuron-to-neuron communication. Golgi staining confirmed changes in synaptic spines compared to control animals. A similar phenotype was observed after exposure of neurons to blood-borne factors due to endothelial cell ablation in the absence of a mechanical impact, suggesting that entrance of blood-borne factors into the brain is sufficient to induce this atypical response. Finally, we found that these “atypical neurons” appear within 5 min after mTBI and remain present for up to 6 months. Surprisingly, we found no evidence of neuronal cell death in areas with BBB leakage even 6 months after mTBI. Thus, this study demonstrates that exposure of neurons to blood-borne factors caused a rapid and sustained loss of neuronal proteins and changes in spine morphology in the absence of neurodegeneration, a finding that is likely relevant to many neuropathologies.

## Materials and Methods

### Animals

Ten to sixteen weeks old C57Bl/6 mice of both sexes were used for mTBI. Mice were bred in-house and breeders were purchased either from The Jackson Laboratory (JAX #000664) or Taconic Biosciences (Taconic C57BL/6NTac).

For endothelial cell-specific ablation, Gt(ROSA)26Sortm1 (DTA)Jpmb/J mice (JAX #006331) were crossed with Tg(Cdh5-cre/ERT2)1Rha (MGI:3848982). We refer to Gt(ROSA)26Sortm1(DTA)Jpmb/J mice that express the Diphtheria Toxin A (DTA) subunit heterozygously as DTA^fl/wt^ mice and Tg(Cdh5-cre/ERT2)1Rha that express the transgene heterozygously as Cdh5(PAC)-CreERT2. To drive Cre expression in adult mice, tamoxifen (10 mg/mL in corn oil) was administered to adult mice via oral gavage at 8.3 g/μL.

Colonies were maintained in a standard pathogen restricted barrier animal facility in groups of five animals at maximum on a 12 h light/12 h dark cycle in the Fralin Biomedical Research Institute (Virginia Tech) animal facility. Humidity and temperature were constant (22°C), with food and water provided *ad libitum*. After all procedures, animals were housed either alone or with littermates that were a part of the same experimental condition until the desired endpoint was reached.

All animal procedures were approved and conducted according to the guidelines of the Institutional Animal Care and Use Committee of Virginia Polytechnic and State University and were done in compliance with the National Institute of Health’s Guide for the Care and Use of Laboratory Animals.

### Mild Single Hit Weight-Drop TBI

We used an impact acceleration model that induced mTBI, which was extensively characterized previously with regard to injury severity, histology, and relevant biological variables, such as loss of consciousness time (Abd-Elfattah Foda and Marmarou, 1994; Marmarou et al., 1994; Nichols et al., 2016; Shandra et al., 2019). Mice 10–16 weeks of age were deeply anesthetized via intraperitoneal injection of ketamine (100 mg/kg)/xylazine (10 mg/kg). Once unconscious, the animal was placed on a foam pad and a flat steel disk was placed on top of the head to diffuse the injury impact throughout the brain. The mouse on the foam was placed under a plexiglass tube containing a 100 g weight, which was dropped from 50 cm height. After the impact, animals immediately underwent transcranial perfusion with Phospho-Buffered Saline (PBS) followed by 4% Paraformaldehyde (PFA). Shams underwent the same procedure except for the impact. The duration between injury and transcardial perfusion was equal to or less than 5 min (⋜5 mpi) for all single hit mTBI (1x TBI). To assess BBB disruption, we used cadaverine conjugated to AlexaFluor-555 (Thermo Fisher Scientific, A30677) which was injected retro-orbitally (0.33 mg/mouse in 100 μL sterile saline) 5 min after ketamine/xylazine injection. Five minutes after cadaverine injection animals underwent TBI/Sham procedure.

### Mild Repetitive Weight-Drop TBI

We used the same impact acceleration model as described above to induce repeated mTBI (3x TBI). Mice 10–16 weeks old were anesthetized with 3.5% isoflurane gas for 5 min and then placed on a foam pad after a subcutaneous administration of the analgesic buprenorphine (0.05–0.1 mg/kg). Injuries were repeated two more times with 45-min intervals, resulting in a total of three injuries. After each injury the mouse was placed on its back on top of a heating pad until consciousness was recovered. Animals were monitored and perfused at either 7 days post-injury (dpi) or 6 months post-injury (mopi). To assess BBB disruptions, mice were retro-orbitally injected with cadaverine under 3% isoflurane anesthesia. Mice were then deeply anesthetized via intraperitoneal injection of ketamine (100 mg/kg)/xylazine (10 mg/kg) and transcardially perfused 30 min after cadaverine conjugated to AlexaFluor-555 (Thermo Fisher Scientific, A30677) was injected.

### Immunohistochemistry

Mice were deeply anesthetized with ketamine (100 mg/kg)/ xylazine (10 mg/kg) and then transcardially perfused with Phospho-Buffered Saline (PBS) followed by 4% Paraformaldehyde (PFA). Tissue was collected and post-fixed overnight and tissue was cut coronally at 50 μm thickness. All primary and secondary antibodies as well as dyes used are all listed in Table 1. Immunochemistry was done according to standard immunohistochemistry protocols used in previous works (Shandra et al., 2019). Antigen retrieval was used for CaMKII and Homer1 before the primary antibody step using 1x Citrate pH 6.0 (Life Technologies) for 5 min at 95°C. Slices were then washed in H_2_O for 10 min and then washed two times in Triton-PBS for 10 min. Then slices were incubated in the primary antibody solution for 24–48 h at 4°C. Imaging of the mouse brain slices was done using a NikonA1R confocal microscope with Apo 40x and 60x oil immersion objectives. We imaged cortical layers II-VI throughout the entire cortex excluding the piriform cortex for all analysis. Layer I was excluded because of low neuronal densities in this area. Where diffuse injury/atypical astrocytes occur within the cortex is unpredictable (Shandra et al., 2019; George et al., 2021). Thus, areas of interest were selected based on reduced Glt1 staining (indicative of atypical astrocytes) and imaged an equivalent area in sham controls.

**TABLE 1:** List of antibodies and dyes.

Name	Manufacturer	Catalog #	RRID	Species raised in	Monoclonal/Polyclonal	Concentration
**Primary antibodies**						
CaMKII	Abcam	AB131468	AB_11157799	Rabbit	Polyclonal	1:200
Glt1	Millipore	AB1783	AB_90949	Guinea pig	Polyclonal	1 : 1,000
Homer1	Synaptic Systems	160 003	AB_887730	Rabbit	Polyclonal	1:500
NeuN	Millipore	ABN78	AB_10807945	Rabbit	Polyclonal	1:1,000
NeuN	Millipore	MAB377	AB_2298767	Mouse	Monoclonal	1:1,000
Parvalbumin	Millipore	MAB1572	AB_2174013	Mouse	Monoclonal	1:1,000
VGLUT1	Millipore	AB5905	AB_2301751	Guinea pig	Polyclonal	1:1,000
**Secondary antibodies**						
Guinea pig Alexa-647	Jackson Immuno Research	106-606-003	AB_2337449	Goat	Polyclonal	1:1,000
Mouse Alexa-488	Jackson Immuno Research	115-546-003	AB_2338859	Goat	Polyclonal	1:1,000
Mouse CY3	Jackson Immuno Research	115-166-003	AB_2338699	Goat	Polyclonal	1:1,000
Rabbit Alexa-488	Jackson Immuno Research	111-546-144	AB_2338057	Goat	Polyclonal	1:1,000
Rabbit CY3	Jackson Immuno Research	111-166-144	AB_2338011	Goat	Polyclonal	1:1,000
**Dyes**						
Alexa-555 Cadaverine	Thermo Fisher Scientific	A30677	N/A	N/A	N/A	0.33 mg/mouse
DAPI	Thermo Fisher Scientific	D1306	AB_2629482	N/A	N/A	1:1,000
NeuroTrace^™^ 530/615 Red Fluorescent Nissl Stain	Thermo Fisher Scientific	N21482	AB_2620170	N/A	N/A	1:100

### Golgi Staining

A sliceGolgi kit (Bioenno, Cat#003760) was used according to the manufacturer’s instructions. In short, 7 dpi mice were deeply anesthetized via intraperitoneal administration of ketamine (100 mg/kg)/xylazine (10 mg/kg) and then were transcardially perfused with 0.9% saline buffer followed by a mixture of dH_2_O (45%), solution A1 (25%), A2 (25%), and A3 (5%) for 25 min. Tissue was collected and post-fixed for 1 h in fixative. Brains were sliced at 50 μm in 0.1 M phosphate buffer (also used to store tissues at 4^°^C). After 1 h post-fixation, brain slices were impregnated in solution B for 72 h and then stained for 2 min with Solution C and post-stained for 1.5 min in solution D. Peroxidase blockage was induced by submerging slices in H_2_O_2_ (0.09% H_2_O_2_ in 10 mL PBS-T) for 20 min. Antigen retrieval used EDTA-HCl (10 mM Tris base, 1 mM EDTA solution, pH9.0) at 95^°^C for 10 min. After cooling and washing in PBS-T, slices were blocked in 5% FBS/ 1% BSA blocking solution for 20 min and were incubated in primary guinea pig anti-Glt1 antibody 1:500 in the blocking solution overnight. On the following day, after three washes in PBS-T, the slices were incubated in anti-guinea pig biotinylated secondary antibody (1:1,000) for 45 min. After three washes, slices were incubated in ABC reagent (VECTASTAIN Elite ABC-HRP Kit, Vector laboratories) for 30 min, washed and incubated in 3,3′ diaminobenzidine (DAB) substrate (DAB substrate Kit, Vector laboratories) until the color turned (approximately 5 min). Slices were then mounted using Depex (VectaMount Permanent Mounting Medium, Vector laboratories).

### Image Analysis and Neuronal Quantifications

For analysis, all images were taken using a 40x oil immersion objective. Neuronal cell density for Nissl, NeuN, CamKII+ and PV+ neurons were quantified in cortical areas with reduced Glt1 expression (atypical areas) using ImageJ and the ImageJ cell counter. Image color and pixel parameters for image quantification were initially set using a sham image and stayed consistent throughout all images for both mTBI and sham mice. For mTBI images, a region of interest (ROI) was drawn around areas with reduced Glt1 expression and the total area of Glt1 loss in μm^3^ was calculated by multiplying the value of the ROI by the number of slices used in the image z-stack. This value was then converted into mm^3^ in order to calculate neuronal cell density. After the establishment of an ROI outline, the cell counter was used to count the number of neurons present only in the ROI, excluding the surrounding neurons outside of the ROI from the quantification. The number of cells counted were then divided by the total area of Glt1 loss in mm^3^ to give the total neuronal cell density within the ROI. Cell density for sham brain slices was calculated in a similar fashion, by using an ROI established in a corresponding mTBI brain slice. Separate counts were generated for NeuN+ cells classified as normal, faint, or mislocalized, where mislocalized cells did not show NeuN expression within the nucleus. Cell densities were determined as described above by either including all Nissl+ cells or by counting Nissl+ pyramidal cells based on morphology. For Nissl and NeuN quantifications, 10 shams/controls, 5 ⋜5 mpi, 7 7dpi, and 4 6mopi animals were analyzed. 3–8 ROIs across 3–7 slices were analyzed per animal For CamKII and PV quantification 5 shams/controls, 5 ⋜5 mpi, and 6 7 dpi animals were analyzed. 3–11 ROIs across 3–7 slices were analyzed per animal. In depth information is available in Supplementary Table 1.

### Synapse Quantification

We used a modified protocol of Ippolito and Eroglu (2010) to quantify the synapses within atypical areas. We used vGlut1 as a presynaptic protein and Homer1 as a postsynaptic protein. Tissue was imaged using a 60x oil immersion objective in mTBI brain slices and the corresponding area of the brain was imaged in shams. We used an ImageJ plugin puncta analyzer tool for analysis of synapses. Briefly, we defined a ROI with a set size that was used to quantify areas of synapses in every image. This set ROI could capture 3–6 neurons at a time. Once the ROI was selected, we used the puncta analyzer tool to quantify the presynaptic puncta, postsynaptic puncta and the colocalization between the two. We converted the amount of puncta per area to volume in mm^3^. For vGlut and Homer1 quantification 5 shams/controls, 5 ⋜5 mpi, and 5 7 dpi animals were analyzed. 3–7 ROIs across 3–7 slices were analyzed per animal. In depth information is available in Supplementary Table 1.

### Golgi Synapse Profile Quantification

After Golgi staining, tissue was imaged using an Olympus BX51 microscope equipped with a 60x oil immersion objective to capture pyramidal neuronal cells in the cortex layers II-IV. Pyramidal neurons were identified based on their location (layer III and V), their size and the triangular morphology of their soma. Only cells that presented consistent staining in soma and processes were analyzed. Images of a cell in different planes were taken using a manual z-controller, opened in ImageJ, combined into a single image, and saved as an image sequence file. The image sequence was opened in the program RECONSTRUCT.^1^ A segment of 10 μm of dendrite was selected in the plane in which the spines looked best defined, and the length and width of spines located in that segment were measured using the RECONSTRUCT series options. Spines were classified as described in Risher et al. (2014). Briefly, spines with length > 2 μm were considered filopodia, length < 2 μm were classified long thin, length < 1 μm as thin, length:width ratio < 1 as stubby, width > 0.6 as mushroom and spines with two or more heads, as branched. For Golgi quantification 3 shams and 3 7 dpi animals were analyzed. One to nine cells per slice were analyzed in 3–6 slices. In-depth information is available in Supplementary Table 1.

### Statistical Analysis

Statistics were calculated and data graphed using GraphPad Prism 9 (GraphPad Software). All data was tested for Gaussian distribution using the Kolmogorov–Smirnov (KS) normality test. Before running ANOVA tests, homoscedasticity was tested using the Levene test. Statistical tests were chosen accordingly and are specified in the results section or figure legend. For all analyses excluding Golgi staining analysis statistics were run after averaging per animal. For Golgi staining analysis each point represents a cell. All graphs display standard error of the mean and statistical significance is indicated with ^∗^*p* ≤ 0.05, ^∗∗^*p* ≤ 0.01, ^∗∗∗^*p* ≤ 0.001.

## Results

### Cortex Areas With Atypical Astrocytes Correlate With a Downregulation of Key Neuronal Proteins but Not Neurodegeneration

**FIGURE 1:**
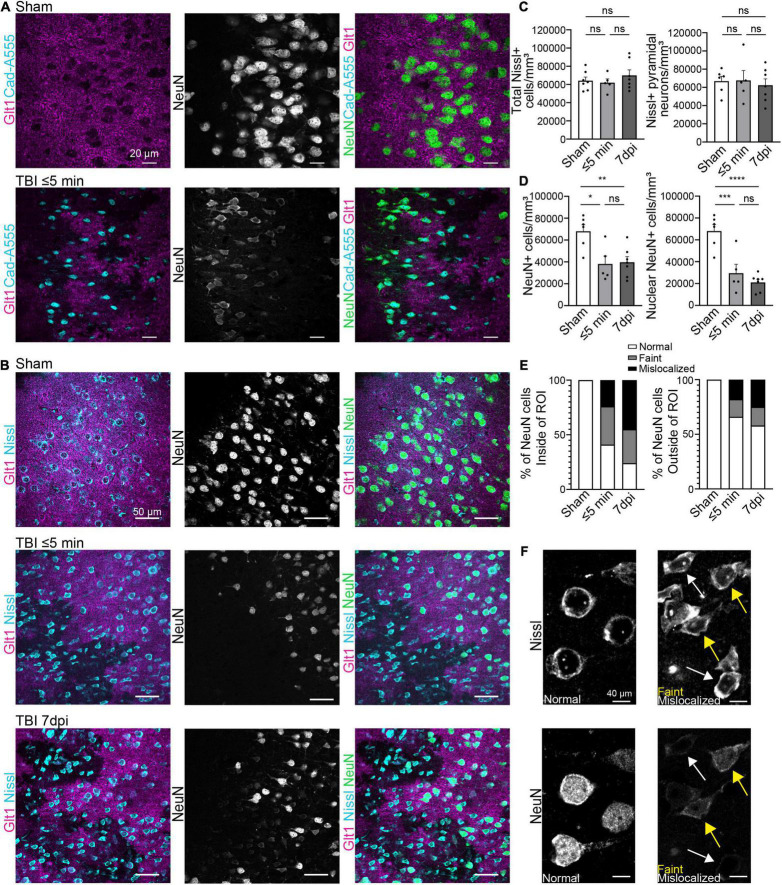
Blood-brain barrier leakage occurs after a single mTBI leading to an atypical NeuN response. **(A)** Cadaverine leakage occurs within 5 min of a single TBI hit and Cadaverine overlaps with regions of atypical neurons. Panels in the top row show Sham and panels on the bottom row are TBI ≤ 5 min (Blue: Cad-A555, Magenta: Glt1, B&W: NeuN, Green: NeuN). **(B)** NeuN expression in mature neurons was reduced in atypical areas within 5 min after a single hit and persisted up to 7 dpi. Nissl staining labeled all pyramidal neurons including neurons with Atypical NeuN expression throughout all time points. Panels in the top row show Sham, panels on the center row are TBI ≤ 5 min and panels on the bottom row are TBI 7 dpi (Blue: Nissl, Magenta: Glt1, B&W: NeuN, Green: NeuN). **(C)** All Nissl expressing cells were quantified as density (cell/mm^3^) in atypical areas and plotted per animal. (Sham: 64,188 ± 3,951, *n* = 7; ≤ 5 min: 61,929 ± 4,186, *n* = 5; 7 dpi: 69,927 ± 6,080, *n* = 7). One-way ANOVA: non-significant, *p*-value = 0.5287. *Post-hoc* analysis (Tukey HSD): Sham vs. ≤ 5 min: *p* = 0.9501; Sham vs. 7 dpi: *p* < 0.6789; ≤ 5 min vs. 7 dpi: *p* = 0.5395. Then only pyramidal Nissl expressing cells were quantified (Sham: 66,887 ± 5,302, *n* = 6; ≤ 5 min: 67,637 ± 10,843, *n* = 5; 7 dpi: 62,390 ± 6,998, *n* = 7) One-way ANOVA: non-significant, *p*-value = 0.8660. *Post-hoc* analysis (Tukey HSD): Sham vs. ≤ 5 min: *p* = 0.9976; Sham vs. 7 dpi: *p* = 0.9029; ≤ 5 min vs. 7 dpi: *p* = 0.8822. **(D)** All NeuN+ cells and NeuN+ nucleus were quantified as density (cell/mm^3^) in regions of atypical astrocytes and plotted per animal. Statistics for all NeuN+ cells (Sham: 68,063 ± 6,018, *n* = 6; ≤ 5 min: 38,112 ± 7,231, *n* = 5; 7 dpi: 39,727 ± 5,131, *n* = 7). One-way ANOVA: significant, *p*-value = 0.0047. *Post-hoc* analysis (Tukey HSD): Sham vs. ≤ 5 min: *p* < 0.0111; Sham vs. 7 dpi: *p* < 0.0091; ≤ 5 min vs. 7 dpi: *p* < 0.9808. Statistics for nuclear NeuN+ cells (Sham: 68,063 ± 6,018, *n* = 6; ≤ 5 min: 29,466 ± 8,146, *n* = 5; 7 dpi: 21,056 ± 2,864, *n* = 7). One-way ANOVA: significant, *p*-value ≤ 0.0001. *Post-hoc* analysis (Tukey HSD): Sham vs. ≤ 5 min: *p* < 0.0008; Sham vs. 7 dpi: *p* < 0.0001; ≤ 5 min vs. 7 dpi: *p* < 0.5527. **(E)** Three different patterns of NeuN expression were observed when quantifying NeuN. Normal expression of NeuN showed consistent bright and vibrant expression in the nucleus of the neuron. Faint expression of NeuN showed a decrease of expression in the Nucleus of the neuron. Mislocalized expression of NeuN show little to no expression of NeuN in the nucleus but expression within the cytoplasm of the cell. Cells were quantified in regions of atypical astrocytes and in neighboring healthy astrocytes and graphed. Inside ROI’s Sham’s cells were 100% normal neurons, in ≤ 5 min cells were 41% Normal, 35% faint, and 24% mislocalized. In 7 dpi cells were 24% Normal, 31% faint, and 45% mislocalized. Outside ROI’s Sham’s cells were 100% normal neurons. In ≤ 5 min cells were 66% Normal, 16% faint, and 18% mislocalized. In 7 dpi cells were 58% Normal, 17% faint, and 25% mislocalized. **(F)** Magnified image of “normal,” “faint,” and “mislocalized” NeuN expression and the corresponding Nissl images. Yellow arrowheads point to faint NeuN expressing neurons and white arrowheads point to mislocalized neurons. *p-value ≤ 0.05, **p-value ≤ 0.01, ***p-value ≤ 0.001, ****p-value ≤ 0.0001.

**FIGURE 2:**
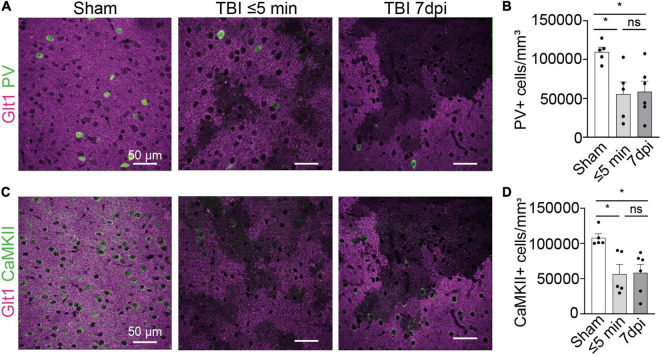
Interneuron and glutamatergic densities changed in areas of atypical astrocytes. **(A)** In atypical areas, expression of the interneuron protein parvalbumin (PV) was reduced. Panel on the left show Sham, panel in the center show TBI ≤ 5 min and panel on the right show TBI 7 dpi (Magenta: Glt1, Green: PV). **(B)** Quantification of PV cell densities (cell/mm^3^) in atypical areas and was plotted per animal and showed on average a reduction of half on. (Sham: 10,956 ± 618.4, *n* = 5; ≤ 5 min: 5,578 ± 1,541, *n* = 5; 7 dpi: 5,716 ± 1,579, *n* = 6). One-way ANOVA: significant, *p*-value = 0.0269. *Post-hoc* analysis (TukeyHSD): Sham vs. ≤ 5 min: *p* < 0.05; Sham vs. 7 dpi *p* < 0.05; ≤ 5 min vs. 7 dpi *p* < 0.9971. **(C)** In atypical areas, expression of the glutamatergic protein CamKII was reduced. Panel on the left show Sham, panel in the center show TBI ≤ 5 min and panel on the right show TBI 7 dpi (Magenta: Glt1, Green: PV). **(D)** Quantification of CamKII cell densities (cell/mm^3^) in atypical areas was plotted per animal and showed on average a reduction of half. (Sham: 107,854 ± 5,644, *n* = 5; ≤ 5 min: 56,743 ± 13,383, *n* = 5; 7 dpi: 64,131 ± 11,321, *n* = 6). One-way ANOVA: significant, *p*-value = 0.0125. *Post-hoc* analysis (Tukey HSD): Sham vs. ≤ 5 min: *p* < 0.05; Sham vs. 7 dpi *p* < 0.05; ≤ 5 min vs. 7 dpi *p* < 0.8773. *p-value ≤ 0.05.

To assess how neurons respond to leakage of blood-borne factors after mTBI, we used retro-orbital injections of cadaverine linked to AlexaFluor-555 to label areas with BBB damage (Armulik et al., 2010; Mizee et al., 2013). To assess if neurons responded immediately to blood-borne factors, brain tissue was harvested within 5 min post-injury (mpi). We used the pan-neuronal protein NeuN, which is widely used in the literature, to quantify neurons. In areas of the cortex where cadaverine labeled neurons indicated BBB disruption, Glt1 protein expression was reduced reproducing our previous findings (Figure 1A). These areas with atypical astrocytes and BBB leakage are from here on named “atypical areas.” In these areas, NeuN was also lost (Figure 2A).

To assess if NeuN loss was due to neuronal death immediately after single or repeated mTBI, immunohistochemistry for Glt-1, NeuN, and the cell dye NeuroTracer (Nissl staining) was performed. Despite the loss of Glt1 and NeuN, there was no change in cell density (Figures 1B,C) (Sham: 64,188 cell/mm^3^ ± 3,951, *n* = 7; ≤ 5 min: 61,929 cell/mm^3^ ± 4,186, *n* = 5; 7 dpi: 69,927 cell/mm^3^ ± 6,080, *n* = 7. One-way ANOVA: non-significant, *p*-value = 0.5287. *Post-hoc* analysis (Tukey HSD): Sham vs. ≤ 5 min: *p* = 0.9501; Sham vs. 7 dpi: *p* < 0.6789; ≤ 5 min vs. 7 dpi: *p* = 0.5395) or pyramidal neuron density (Figures 1B,D) (Sham: 66,887 cell/mm^3^ ± 5,302, *n* = 6; ≤ 5 min: 67,637 cell/mm^3^ ± 10,843, *n* = 5; 7 dpi: 62,390 cell/mm^3^ ± 6,998, *n* = 7). One-way ANOVA: non-significant, *p*-value = 0.8660. *Post-hoc* analysis (Tukey HSD): Sham vs. ≤ 5 min: *p* = 0.9976; Sham vs. 7 dpi: *p* = 0.9029; ≤ 5 min vs. 7 dpi: *p* = 0.8822) suggesting that neurons remained alive after their contact with blood-borne factors. Yet, there was a reduction in the number of neurons expressing NeuN ⋜5 mpi but, we found no difference in NeuN+ cell density 7 days post-injury (dpi) (Figures 1B,D) (Sham: 68,063 cell/mm^3^ ± 6,018, *n* = 6; ≤ 5 min: 38,112 cell/mm^3^ ± 7,231, *n* = 5; 7 dpi: 39,727 cell/mm^3^ ± 5,131, *n* = 7. One-way ANOVA: significant, *p*-value = 0.0047. *Post-hoc* analysis (Tukey HSD): Sham vs. ≤ 5 min: *p* < 0.0111; Sham vs. 7 dpi: *p* < 0.0091; ≤ 5 min vs. 7 dpi: *p* < 0.9808). However, in atypical areas, more than 60% of NeuN+ neurons had a mislocalized staining of NeuN within the cytoplasm but not the nucleus or a faint staining both at ⋜5 mpi or 7 dpi (Figures 1E–F) (Sham: 68,063 cell/mm^3^ ± 6,018, *n* = 6; ≤ 5 min: 29,466 cell/mm^3^ ± 8,146, *n* = 5; 7 dpi: 21,056 cell/mm^3^ ± 2,864, *n* = 7. One-way ANOVA: significant, *p-*value ≤ 0.0001. *Post-hoc* analysis (Tukey HSD): Sham vs. ≤ 5 min: *p* < 0.0008; Sham vs. 7 dpi: *p* < 0.0001; ≤ 5 min vs. 7 dpi: *p* < 0.5527). This did not occur outside of areas with BBB leakage (Figure 1E). NeuN was normally localized inside the nucleus in 41% of all NeuN+ neurons at ⋜5 mpi and in 24% of the NeuN+ cells at 7 dpi (Figures 1D,E). NeuN is a splicing regulator factor that needs to be located in the nucleus to properly function and neurocognitive deficits have been observed when this function is disrupted. Thus, NeuN mislocalization after mTBI may have functional implications.

We next assessed the expression of other neuronal proteins in atypical areas. We stained for the interneuron protein parvalbumin (PV). In atypical areas, the density of PV+ neurons was decreased by half both at ⋜5 mpi and 7 dpi (Figures 2A,B) (Sham: 10,956 cells/mm^3^ ± 618.4, *n* = 5; ≤ 5 min: 5,578 cells/mm^3^ ± 1,541, *n* = 5; 7 dpi: 5,716 cells/mm^3^ ± 1,579, *n* = 6. One-way ANOVA: significant, *p*-value = 0.0269. *Post-hoc* analysis (TukeyHSD): Sham vs. ≤ 5 min: *p* < 0.05; Sham vs. 7 dpi *p* < 0.05; ≤ 5 min vs. 7 dpi *p* < 0.9971). Similarly, the density of CaMKII+ glutamatergic neurons was reduced by half at ⋜5 mpi and 7 dpi (Figures 2C,D) (Sham: 107,854 cells/mm^3^ ± 5,644, *n* = 5; ≤ 5 min: 56,743 cells/mm^3^ ± 13,383, *n* = 5; 7 dpi: 64,131 cells/mm^3^ ± 11,321, *n* = 6. One-way ANOVA: significant, *p*-value = 0.0125. *Post-hoc* analysis (Tukey HSD): Sham vs. ≤ 5 min: *p* < 0.05; Sham vs. 7 dpi *p* < 0.05; ≤ 5 min vs. 7 dpi *p* < 0.8773). These results indicate a loss of several neuronal proteins immediately after mTBI in areas of BBB disruption. Because of the striking similarities of this phenotype to atypical astrocytes, we named these neurons within areas with BBB leakage characterized by abnormal or reduced protein expression “atypical neurons.”

### Synapses Are Altered in Atypical Neurons

**FIGURE 3:**
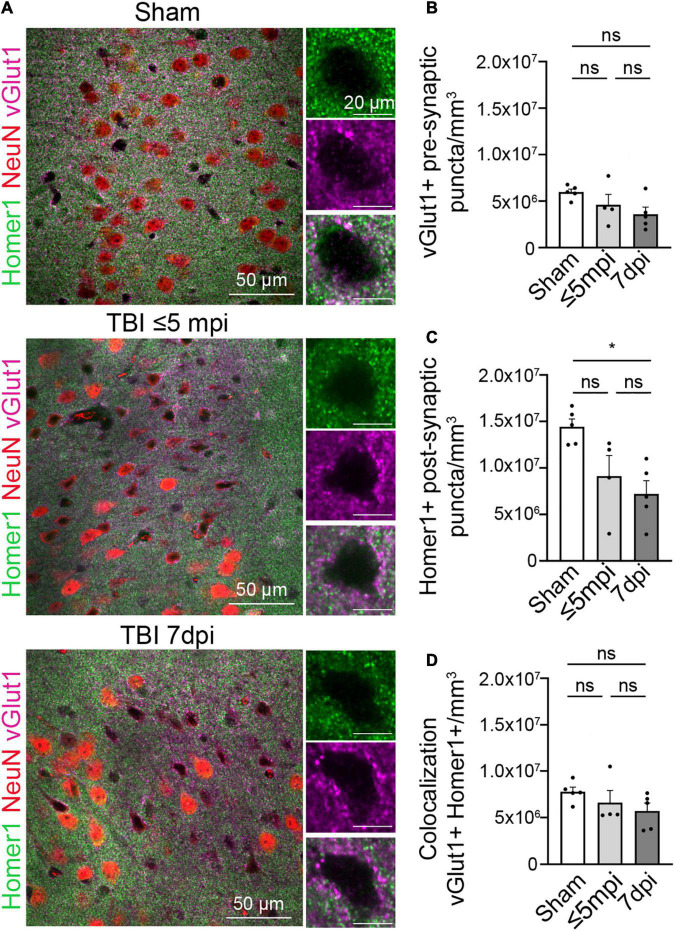
Postsynaptic proteins are altered in atypical areas. **(A)** Neurons with atypical NeuN expression show a decrease in the postsynaptic Homer1 protein while the presynaptic protein vGlut1 expression remains constant. Each panel consisted of a large image of an area of interest and to the right side of the image shows 1 cell located in the area of interest. Panels on the top row show Sham, panel in the center row show TBI ≤ 5 min and panel on the bottom row show TBI 7 dpi (Magenta: vGlut1, Red: NeuN, Green: Homer1). **(B)** Number of vGlut1 puncta in a density of 3–5 cells (puncta/mm^3^) was quantified and plotted per animal. There was no change in densities among all timepoints (Sham, 7,767,199 ± 500,109, *n* = 5; ≤ 5 min, 6,604,101 ± 1,297,827, *n* = 4; 7 dpi, 5,713,619 ± 843,873, *n* = 5). One-way ANOVA: non-significant, *p*-value = 0.2703. *Post-hoc* analysis (Tukey HSD): Sham vs. ≤ 5 min: *p* < 0.6422; Sham vs. 7 dpi: *p* < 0.2433; ≤ 5 min vs. 7 dpi: *p* < 0.7679. **(C)** Number of Home1 puncta in a density of 3–5 cells(puncta/mm^3^) was quantified and plotted per animal. There was no significant change in densities between sham and ≤ 5 min, but there was a significant change between sham and 7 dpi. (Sham, 14,424,739 ± 835,170, *n* = 5; ≤ 5 min, 9,124,708 ± 2,214,359, *n* = 4; 7 dpi, 7,205,630 ± 1,414,819, *n* = 5) One-way ANOVA: significant, *p*-value = 0.0122. *Post-hoc* analysis (Tukey HSD): Sham vs. ≤ 5 min: *p* < 0.0742; Sham vs. 7 dpi: *p* < 0.05; ≤ 5 min vs. 7 dpi: *p* < 0.6554. **(D)** Number of colocalized puncta between homer1 and vGlut1 in a density of 3–5 cells (puncta/mm^3^) was quantified and plotted per animal. There was no change in densities among all timepoints (Sham, 5,971,345 ± 324,190, *n* = 5; ≤ 5 min, 4,603,244 ± 1,127,077, *n* = 4; 7 dpi, 3,598,635 ± 758,416, *n* = 5). One-way ANOVA: non-significant, *p*-value = 0.1111. *Post-hoc* analysis (Tukey HSD): Sham vs. ≤ 5 min *p* < 0.4453; Sham vs. 7 dpi *p* < 0.0952; ≤ 5 min vs. 7 dpi *p* < 0.6367. *p-value ≤ 0.05.

Since synaptic contacts are essential to normal neuronal function, we next evaluated the synaptic density within atypical areas. We performed immunohistochemistry for the excitatory presynaptic protein vGlut1 and the postsynaptic protein Homer1. We then determined the number of presynaptic and postsynaptic puncta and colocalization between both, which is indicative of synapses. Presynaptic vGlut1+ puncta were unchanged in atypical areas after mTBI when compared to shams, ⋜5 mpi, and 7 dpi (Figures 3A,B) (Sham, 7,767,199 puncta/mm^3^ ± 500,109, *n* = 5; ≤ 5 min, 6,604,101 puncta/mm^3^ ± 1,297,827, *n* = 4; 7 dpi, 5,713,619 puncta/mm^3^ ± 843,873, *n* = 5). One-way ANOVA: non-significant, *p*-value = 0.2703. *Post-hoc* analysis (Tukey HSD): Sham vs. ≤ 5 min: *p* < 0.6422; Sham vs. 7 dpi: *p* < 0.2433; ≤ 5 min vs. 7 dpi: *p* < 0.7679). However, there was a decrease in the number of Homer1+ puncta at ⋜5 mpi and 7 dpi in atypical areas (Figures 3A,C) (Sham, 14,424,739 puncta/mm^3^ ± 835,170, *n* = 5; ≤ 5 min, 9,124,708 puncta/mm^3^ ± 2,214,359, *n* = 4; 7 dpi, 7,205,630 puncta/mm^3^ ± 1,414,819, *n* = 5. One-way ANOVA: significant, *p*-value = 0.0122. *Post-hoc* analysis (Tukey HSD): Sham vs. ≤ 5 min: *p* < 0.0742; Sham vs. 7 dpi: *p* < 0.05; ≤ 5 min vs. 7 dpi: *p* < 0.6554). Yet, we found that ⋜5 mpi and 7 dpi animals showed no changes in vGlut1+/Homer1+ colocalized puncta (Figures 3A,D) (Sham, 5,971,345 puncta/mm^3^ ± 324,190, *n* = 5; ≤ 5 min, 4,603,244 puncta/mm^3^ ± 1,127,077, *n* = 4; 7 dpi, 3,598,635 puncta/mm^3^ ± 758,416, *n* = 5. One-way ANOVA: non-significant, *p-*value = 0.1111. *Post-hoc* analysis (Tukey HSD): Sham vs. ≤ 5 min *p* < 0.4453; Sham vs. 7 dpi *p* < 0.0952; ≤ 5 min vs. 7 dpi *p* < 0.6367). This result may point to a decrease in the synaptic connections between the atypical neurons and other neurons or could be due to loss of Homer1 protein similar to the loss of other neuronal proteins in atypical areas while postsynaptic spines remain in place.

**FIGURE 4:**
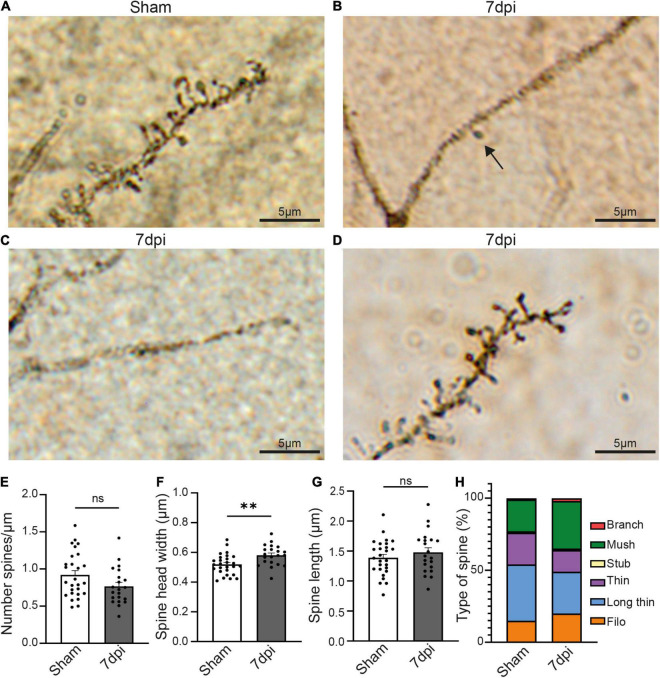
mTBI induces changes in pyramidal neurons’ synaptic spines. **(A)** Representative Golgi staining of synaptic spines in the pyramidal cell of a sham animal. **(B)** Representative Golgi staining of a dendrite of a pyramidal cell where only one spine can be visualized (black arrow) 7 dpi animal. **(C)** Representative Golgi staining of a pyramidal cell in a 7 dpi animal, in which dendritic spines cannot be visualized. **(D)** Golgi staining of dendritic spines of a pyramidal cell in which dendritic spines are visible 7 dpi. **(E)** Spine density (number of spine/μm) in dendrites where spines were visible. Each dot represents a cell (Sham: 0.9207 ± 0.05819, *n* = 27 cells, 3 animals; 7 dpi: 0.7677 ± 0.05374, *n* = 21 cells, 3 animals). Two-tailed *t*-test: non-significant, *p*-value = 0.0660. **(F)** Spine head width (μm) in dendrites where spines were visible. Each dot represents a cell (Sham: 0.5194 ± 0.01329, *n* = 27 cells, 3 animals; 7 dpi: 0.5811 ± 0.06883, *n* = 21 cells, 3 animals). Two-tailed *t*-test: significant, *p*-value = 0.0035 (**p-value ≤ 0.01). **(G)** Spine length (μm) in dendrites where spines were visible. Each dot represents a cell (Sham: 1.388 ± 0.05715, *n* = 27 cells, 3 animals; 7 dpi: 1.480 ± 0.07562, *n* = 21 cells, 3 animals). Two-tailed *t*-test: non-significant, *p*-value = 0.3310. **(H)** Descriptive graphic with the percentage of each spine type (red: branch, green: mush, yellow: stub, purple: thin, blue: long thin, orange: filo) in sham and 7 dpi animals.

To distinguish between these possibilities, we next quantified spine number and morphology using a classic Golgi staining. Unfortunately, the fixation and impregnation process of the Golgi staining was incompatible with Glt1 DAB staining. We thus were not able to stratify atypical areas. Overall, the density of pyramidal neurons stained with Golgi was reduced in 7 dpi mTBI compared to shams. Within the Golgi+ pyramidal neurons, we observed some cells with no or almost no spines (Figures 4B,C). Yet, other mTBI pyramidal neurons had spines. When we analyzed this latter subset of pyramidal cells that did have spines, we found that synaptic density in cortical pyramidal cells was unchanged at 7 dpi when compared to shams (Figures 4A,D) (Sham: 0.9207 spine/μm ± 0.05819, *n* = 27 cells, 3 animals, 8–11 cells per animal; 7 dpi: 0.7677 spine/μm ± 0.05374, *n* = 21 cells, 3 animals, 5–13 cells per animal. Two-tailed *t*-test: non-significant, *p*-value = 0.0660). However, spine heads were wider (Figure 4E) (Sham: 0.5194 μm ± 0.01329, *n* = 27 cells, 3 animals, 8–11 cells per animal; 7 dpi: 0.5811 μm ± 0.06883, *n* = 21 cells, 3 animals. Two-tailed *t*-test: significant, *p*-value = 0.0035) while spine length was unchanged (Figure 4F) (Sham: 1.388 μm ± 0.05715, *n* = 27 cells, 3 animals; 7 dpi: 1.480 μm ± 0.07562, *n* = 21 cells, 3 animals, 5–13 cells per animal. Two-tailed *t*-test: non-significant, *p*-value = 0.3310).

Changes in the type of spine is indicative of synaptic plasticity with larger spine heads suggestive of increased synaptic strength. We classified the spine types based on their width/length ratio as previously described (Risher et al., 2014). In sham animals, the major spine type was long-thin (39% of the total of spines), followed by thin and mushroom (22% each), filopodia (15%), stubby and branched (1% each). Yet, after mTBI, the most frequent type of spine was mushroom (33%), followed by long-thin (29%), filopodia (20%), thin (15%), branched (2%) and stubby (1%) (Figure 4G). These results suggest that mTBI induces a heterogeneous response in different pyramidal neurons, in which some of them have little or no Golgi+ spines whereas others have no changes in density but alterations of the structure of the synaptic spines.

### Atypical Neurons Are Induced by Blood-Borne Factors

**FIGURE 5:**
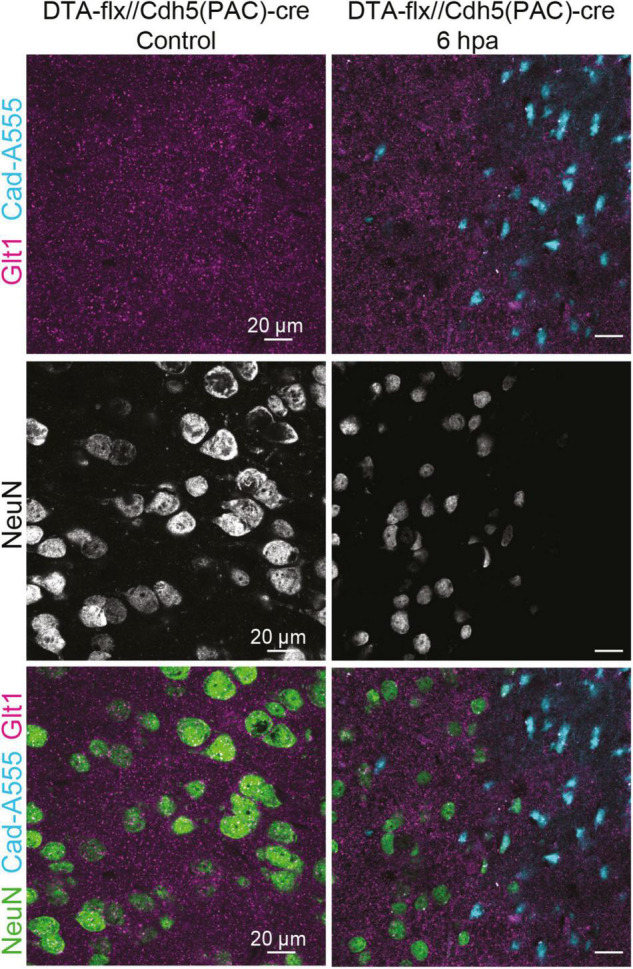
Cadaverine leakage induced by endothelial-cell ablation is sufficient to trigger atypical NeuN expression without a mTBI. Representative images of the control are located on the left and 6 h post Endothelial-cell ablation images are located on the right (Blue: Cad-A555, Magenta: Glt1, B&W: NeuN, Green: NeuN).

To assess if the entrance of blood-borne factors into the brain is sufficient to cause the atypical neuronal phenotype in the absence of other mechanical injury caused by diffuse TBI, we induced BBB damage by sparse genetic ablation of endothelial cells. This was achieved by crossing mice expressing a conditional allele of the diphtheria subunit A (DTA^(fl/wt)^) and endothelial cell specific Cdh5(PAC)-CreERT2 mice. Adult progeny were injected with a single dose of tamoxifen, inducing the apoptosis of endothelial cells because DTA inhibits protein synthesis (this subunit alone is not toxic). Control animals had either an incomplete genotype (lacking one of the two alleles necessary to induce cell ablation) or corn oil was administered instead of tamoxifen. The apoptosis of endothelial cells in blood vessels led to leakage of blood-borne substances, which was assessed by the presence of cadaverine, conjugated with AlexaFluor-555, in the brain parenchyma (Figure 5). Due to the low dosage of the tamoxifen, the ablation was sparse (George et al., 2021).

While assessing NeuN staining in the areas where cadaverine leakage was present, we observed the same neuronal phenotype that had been observed in areas with mTBI-induced atypical areas: mislocalization, decrease, or complete loss of NeuN (Figure 5). This result demonstrates that exposure to blood-borne factors is sufficient to cause atypical neurons in the absence of other mechanical injury such as diffuse axonal damage.

### Atypical Neuron Phenotype Is Maintained Chronically

**FIGURE 6:**
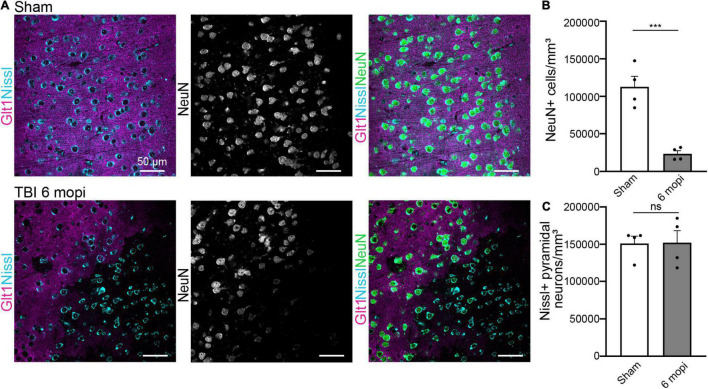
Atypical NeuN expression is maintained 6 months after injury. **(A)** The reduction in atypical NeuN expression in atypical areas persisted up to 6 mopi, while Nissl continues to stain all NeuN expressing subtypes. Panels in the top row show Sham, panels on and panels on the bottom row are TBI 6 mopi (Blue: Nissl, Magenta: Glt1, B&W: NeuN, Green: NeuN). **(B)** NeuN cell density (cell/mm^3^) was measured in atypical areas 6 mopi and plotted per animal (Sham, 112,677 ± 14,024, *n* = 4; 6 mopi, 23,311 ± 4,056, *n* = 4). Two-tailed *t*-test: significant, *p* ≤ 0.001. **(C)** Nissl cell density (cell/mm^3^) was measured in atypical areas 6 mopi and plotted per animal (Sham, 151,096 ± 9,742, *n* = 4; 6 mopi, 152,232 ± 16,018, *n* = 4). Two-tailed *t*-test: non-significant, *p* ≤ 0.9537. *p-value ≤ 0.05, **p-value ≤ 0.01, ***p-value ≤ 0.001, ****p-value ≤ 0.0001.

We next assessed if this phenotype was maintained or resolved with time and if it had long-term consequences for cell survival 6 months post injury (6 mopi). Neuronal densities were assessed using both Nissl and NeuN. Nissl+ neuronal density did not change in 6 mopi animals. (Sham, 151,096 cell/mm^3^ ± 9,742, *n* = 4; 6 mopi, 152,232 cell/mm^3^ ± 16,018, *n* = 4. Two-tailed *t*-test: non-significant, *p* ≤ 0.9537). However, mislocalization or loss of NeuN persisted in atypical areas (Figures 6A,B) (Sham, 112,677 cell/mm^3^ ± 14,024, *n* = 4; 6 mopi, 23,311 cell/mm^3^ ± 4,056, *n* = 4. Two-tailed *t*-test: significant, *p* ≤ 0.001). These results imply that the early atypical neuron phenotype is maintained up to 6 months after the injury, suggesting that this early event could have long term consequences.

## Discussion

There is increasing evidence of the long-term consequences of cTBI in patients and animal models. Yet, the mechanisms underlying these brain changes are not fully understood. BBB disruption is a primary injury that has been reported at all injury severities—mild, moderate, and severe TBI (Wu et al., 2020). Several studies demonstrated a reduction in the entry of blood-borne factors 14 days after focal injury due to glial boundary formation (Bush et al., 1999). Yet, when BBB disruption was measured using IgG infiltration in the brain and microbleeds detected via MRI are sustained for months or even years after the injury in mouse models and patients (Tomkins et al., 2011; Doherty et al., 2016; O’Keeffe et al., 2020). Recent studies have linked microbleeds to worse outcomes after cTBI (Griffin et al., 2019). Yet, the cellular and molecular events triggered by exposure of the brain to blood-borne factors are only starting to be illuminated in this specific pathological context.

In previous studies, we identified blood plasma proteins as the cause of an atypical astrocytic response. Atypical astrocytes rapidly lost all tested astrocyte-typic proteins and lacked expression of classic astrogliosis markers (Shandra et al., 2019; George et al., 2021). Here, we assessed the response of neurons in areas with atypical astrocytes and BBB dysfunction.

### Blood-Brain Barrier Disruption Results in Rapid Appearance of an Atypical Neuronal Phenotype Simultaneously With Atypical Astrocytes

To assess BBB dysfunction we used the polyamine cadaverine conjugated to a fluorophore (AlexaFluor 555), an established marker for BBB dysfunction that labels specifically neurons when it enters the brain parenchyma (Armulik et al., 2010; Mizee et al., 2013). While there are not studies in which the permeability of cadaverine has been assessed, the polar functional groups in its chemical structure and the studies in other polyamines (Gainetdinov et al., 2018) suggest that cadaverine might be impermeable, and therefore, be used as a marker of cell membrane integrity as others have done before (Witte et al., 2019). This is supported by the notion that most cell types (glial cells, endothelial, stromal cells) are not labeled when vessel damage occurs. It has previously been reported that TBI causes neuronal membrane disruption (Witte et al., 2019), which may be the cause for selective uptake of Cadaverine by this cell type upon disruption of the BBB.

The loss of the neuronal markers NeuN, PV and CamKII in areas of BBB disruption occurs within minutes after mTBI, while the cells remain alive. Similarly, atypical astrocytes lose expression of Glt1 and many other proteins within minutes (data here and George et al., 2021). After brain injury, tissue survival is dependent on the ability of cells to rapidly respond to changes in the environment. This likely is one of those responses. There are two aspects to this response: (1) the activation of genes or gene groups to respond to the new conditions (yet to be identified in the context of atypical astrocytes and neurons), and (2) the degradation of proteins that are unnecessary or harmful in the new context. Given that the median half-life of NeuN, parvalbumin, CaMKII and Homer1 is in the range of 2–14 days (Fornasiero et al., 2018) and loss of these proteins occurs within minutes, active degradation rather than protein decay is likely involved. Proteins can be individually and selectively degraded (Cooper, 2000). After mTBI, many proteins were lost simultaneously in both neurons and astrocytes, suggesting coordinated degradation. In other organisms and in cell culture, a coordinated degradation response occurs in response to new environmental conditions, but whether it exists in the mammalian brain and how it is regulated is unknown.

### Blood-Brain Barrier Disruption Results in Rapid Appearance of an Atypical Neuronal Phenotype Simultaneously With Atypical Astrocytes

We also analyzed the expression of other neuronal markers to distinguish if this response is specific to NeuN or similar to the atypical astrocyte response that appears unspecific to a range of proteins. Because PV+ inhibitory interneurons are known to be more susceptible to injury (Campbell et al., 2015), we also wanted to assess whether specific neuronal types are susceptible to this protein loss. Reduced numbers of both, PV+ and CaMKII+ neurons, suggest that excitatory and inhibitory neurons respond atypically to mTBI-induced BBB leakage. Because astrocytes and neurons both respond to BBB leakage with a rapid loss of key proteins, we suggest that the trigger mechanism may be shared in both cell types.

### Blood-Borne Factors Are Sufficient to Induce Atypical Neurons

To identify whether blood-borne factors were sufficient to cause atypical neurons in the absence of other mechanical injury induced by mTBI [e.g., diffuse axonal damage (Dunn-Meynell and Levin, 1997), tau mislocalization (Braun et al., 2020), and blood vessel rupture or shearing (Chen et al., 2003; Griffin et al., 2019)], we induced BBB leakage by using an endothelial cell ablation model that causes sparse endothelial cell apoptosis. As a result, entry of small size tracers such as cadaverine occurs but leakage of large blood plasma proteins such as fibrinogen is rare (George et al., 2021). Here, we demonstrate that this disruption of the BBB is sufficient to cause atypical neurons. Interestingly, cadaverine rapidly labels neurons in the absence of a mechanical insult (as early as 2 h after Tamoxifen induction (George et al., 2021), suggesting that blood-borne factors in the brain may be sufficient to induce plasma membrane disruption in neurons. These findings suggest that entry of blood-borne factors into the brain might serve as an underlying mechanism present in not only mTBI cases, but also in aging and neurological disorders in which the BBB is compromised, such as glioma, Alzheimer’s disease, and multiple sclerosis. Yet, the exact blood-borne factor(s) triggering this atypical response in astrocytes and neurons and the triggered pathways remain to be identified.

### Loss of Proteins in Atypical Neurons May Cause Functional Impairments

NeuN is often used as a pan-neuronal marker to quantify neuronal densities, also after TBI. Yet NeuN, also known as RNA-binding Fox3 protein (Rbfox3), has highly relevant biological functions. NeuN/Rbfox3 is a pre-mRNA splicing regulator (Kim et al., 2009). Mutations in NeuN/Rbfox3 are implicated in neurodevelopmental delay (Utami et al., 2014), cognitive impairments (Lucas et al., 2014), symptoms indicative of autism spectrum disorder (Cooper et al., 2011), and seizures (Lal et al., 2013). Rbfox3 deletion is associated with increased synaptic transmission and plasticity and changes in genes implicated in synaptic function (Wang et al., 2015; Lin et al., 2016). After mTBI, we found that half of the neurons in areas with BBB leakage had lost their regular NeuN expression pattern within minutes. One week later 75% of all neurons were affected, likely impacting normal NeuN/Rbfox3 function. Similar changes to NeuN expression or localization had already been noted in other pathologies including stab wound TBI, axon damage, cerebral ischemia, and epilepsy (McPhail et al., 2004; Ünal-Çevik et al., 2004; Robel et al., 2011; Hernandez et al., 2019), yet the functional consequences of these changes are unknown. Also, there are resident NeuN-cortical neurons particularly susceptible to TBI that may be overlooked using NeuN as a neuronal marker. One recent report demonstrates that this NeuN-population is more susceptible to plasma membrane disruption after TBI (Hernandez et al., 2019). Yet, whether the mTBI-induced NeuN- population we have identified is more susceptible to successive damage, as in the case of repeated TBI, has to be evaluated. Based on these data, we caution the use of NeuN for quantification of neuronal densities in pathology, especially in TBI studies.

PV is a Ca^+2^ binding protein present in GABAergic interneurons. PV-dependent Ca^+2^ buffering regulates the amplitude and time course of intracellular Ca^+2^ transients in terminals after an action potential. Deletion of PV leads to an increase in neural facilitation, a type of short-term synaptic plasticity (Caillard et al., 2000). Furthermore, PV expression is reduced in autism spectrum disorder animal models (Wöhr et al., 2015; Filice et al., 2016). Thus, PV loss in half of the neurons in atypical areas might have a functional impact in the neural facilitation in atypical areas.

CaMKII is an abundant Ca^+2^-dependent postsynaptic kinase with a key role in long-term memory formation. CaMKII is critical for the induction of long-term potentiation (LTP) of synaptic transmission and for the formation of synaptic tags and metaplasticity (reviewed in Irvine et al., 2006; Lucchesi et al., 2011). Mutations in this protein are sufficient to induce deficits in spatial memory formation in mice (Elgersma et al., 2004). The reduction of neurons expressing CaMKII by a half after mTBI might therefore be implicated in neuron-neuron communication and have global behavioral consequences (Vigil et al., 2017). Yet, to determine the impact of the loss of these neuronal proteins in the atypical areas following mTBI, additional studies, beyond the scope of this manuscript, are needed.

### Reduction of Postsynaptic Proteins and Changes in Spine Heads After mTBI Indicates Changes in Neuronal Synapses

Apart from assessing classic neuronal markers, we also quantified the changes in excitatory synapses. Whereas the postsynaptic synapse protein Homer1 was reduced, presynaptic terminals labeled with vGlut1 and total synapse numbers were unchanged. Postsynaptic Homer1 may be degraded by the mechanisms suggested above while presynaptic terminals may be unchanged because neurons projecting into atypical areas may not be affected by blood-borne factors. Homer1 is a scaffolding protein involved in the regulation of the spine morphogenesis and stability and the regulation of the glutamatergic synapses (Yoon et al., 2021). Lack of Homer1 expression reshapes the postsynaptic proteome and affects the spine head size (Yoon et al., 2021).

Because reduced Homer1 protein could affect spine structure, we used Golgi staining and assessed spine density and shape. While we were not able to unequivocally distinguish atypical neurons, Golgi staining revealed interesting differences between mTBI and shams. For unclear reasons, there were fewer neurons labeled after mTBI. Of those that could be identified as pyramidal neurons based on their morphology, some had dendrites that lacked spines while others had similar spine densities when compared to shams. It is conceivable that those neurons without spines may be located in atypical areas. If a subset of neurons in atypical areas lack spines, this may also account for reduced Homer1 puncta.

In those neurons that had spines, spine head width was increased 1 week after mTBI, while spine length was unchanged. This translated into changes in relative representations of spine types: mushroom spines with large spine heads were proportionally increased while long thin spines were decreased. Previous studies suggest that Homer1 expression and its interaction with its partner Ankyrin-G favors an enlargement in the spine head (Yoon et al., 2021). Yet, we found the opposite phenomenon after mTBI where reduced Homer1 coincided with enlarged spine heads. Whether other proteins in the Homer family might be compensating for reduced Homer1 levels or whether the increased proportion of enlarged spines is due to a preferential loss of other spine types is yet to be resolved.

There is a direct correlation between the size of the synaptic spine and synaptic strength (Hayashi and Majewska, 2005; Alvarez and Sabatini, 2007; Ebrahimi and Okabe, 2014). Spine morphology shapes the size and duration of Ca^+2^ transients, influencing plasticity at the synapse (Yuste and Denk, 1995). An increase in mushroom spines relative to long and thin spines may suggest more stable synapses and reduced plasticity after mTBI. However, functional experiments need to test this hypothesis.

Changes in spines may also be due to other mechanisms, e.g., fibrinogen deposition in the brain induces microglia-induced spine elimination in an Alzheimer’s disease model (Merlini et al., 2019). While fibrinogen deposition only occurs in very few areas with BBB dysfunction after mTBI (George et al., 2021), we can generally not rule out that parallel mechanisms may contribute to functional changes in neurons. It will be crucial to assess if protein degradation in atypical astrocytes drives changes in spine morphology or whether those may be a consequence of injury-induced synaptic pruning.

### Atypical Neurons Persist for 6 Months After mTBI

Lastly, we investigated whether the atypical neuronal phenotype is resolved over time or may result in neurodegeneration. Given that our observations at 6 months after mTBI are similar to those early after injury, we conclude that the phenotype does not resolve. Unchanged neuronal cell densities point to a survival of these cells despite the long-term loss of proteins including NeuN, PV, CamKII, and Homer1. In George et al. (2021), we demonstrated that leakage of blood-borne factors and atypical astrocytes persist for several months after mTBI. Thus, continuous presence of blood-borne factors may sustain the phenotype. Why the relatively minor BBB damage is not repaired and if such repair would allow for recovery of protein expression remains unknown. In summary, our findings indicate that atypical neuron induction is one of the earliest events following mTBI and this phenotype is chronically maintained over time, with potential long-term consequences for neuronal function.

## Conclusion

The work presented here sheds light on the consequences of mTBI-induced exposure to blood-borne factors on neurons. While BBB breakdown has been suggested to cause neuronal death, and indeed does so in some pathologies, mTBI caused rapid and sustained loss of neuronal proteins in both excitatory and inhibitory neurons, as well as changes in spine morphology and the post-synapse that may contribute to the cognitive symptoms in patients. Yet, neurons in contact with blood-borne factors remained alive for 6 months after the injury, potentially opening avenues for therapeutic intervention.

## Data Availability Statement

The original contributions presented in the study are included in the article/Supplementary Material, further inquiries can be directed to the corresponding author.

## Ethics Statement

The Virginia Tech’s Institutional Animal Care and Use Committee reviewed and approved all these animal studies under protocol #19–136.

## Author Contributions

CM-B, DM, and SR designed and planned the experiments. CM-B, DM, YR, and AK conducted experiments, collected, and analyzed the data. CM-B, DM, YR, AK, and SR reviewed and edited the manuscript. CM-B, DM, and SR wrote the manuscript with input from all authors.

## Conflict of Interest

The authors declare that the research was conducted in the absence of any commercial or financial relationships that could be construed as a potential conflict of interest.

## Publisher’s Note

All claims expressed in this article are solely those of the authors and do not necessarily represent those of their affiliated organizations, or those of the publisher, the editors and the reviewers. Any product that may be evaluated in this article, or claim that may be made by its manufacturer, is not guaranteed or endorsed by the publisher.

## Footnotes

1
https://synapseweb.clm.utexas.edu/software-0

## References

[B1] Abd-Elfattah Foda M. A. Marmarou A. (1994). A new model of diffuse brain injury in rats. Part II: morphological characterization. *J. Neurosurg.* 80 301–313. 10.3171/JNS.1994.80.2.0301 8283270

[B2] Adams R. Schachtrup C. Davalos D. Tsigelny I. Akassoglou K. (2007). Fibrinogen signal transduction as a mediator and therapeutic target in inflammation:lessons from multiple sclerosis. *Curr. Med. Chem.* 14 2925–2936. 10.2174/092986707782360015 18045138

[B3] Alvarez V. A. Sabatini B. L. (2007). Anatomical and physiological plasticity of dendritic spines. *Annu. Rev. Neurosci.* 30 79–97. 10.1146/annurev.neuro.30.051606.094222 17280523

[B4] Armulik A. Genové G. Mäe M. Nisancioglu M. H. Wallgard E. Niaudet C. (2010). Pericytes regulate the blood-brain barrier. *Nature* 468 557–561. 10.1038/nature09522 20944627

[B5] Bramlett H. M. Dietrich W. D. (2015). Long-term consequences of traumatic brain injury: current status of potential mechanisms of injury and neurological outcomes. *J. Neurotrauma* 32 1834–1848. 10.1089/NEU.2014.3352 25158206 PMC4677116

[B6] Braun N. J. Yao K. R. Alford P. W. Liao D. (2020). Mechanical injuries of neurons induce tau mislocalization to dendritic spines and tau-dependent synaptic dysfunction. *Proc. Natl. Acad. Sci. U.S.A.* 117 29069–29079. 10.1073/PNAS.2008306117/-/DCSUPPLEMENTAL 33139536 PMC7682580

[B7] Bush T. G. Puvanachandra N. Horner C. H. Polito A. Ostenfeld T. Svendsen C. N. (1999). Leukocyte infiltration, neuronal degeneration, and neurite outgrowth after ablation of scar-forming, reactive astrocytes in adult transgenic mice. *Neuron* 23 297–308. 10.1016/S0896-6273(00)80781-3 10399936

[B8] Caillard O. Moreno H. Schwaller B. Llano I. Celio M. R. Marty A. (2000). Role of the calcium-binding protein parvalbumin in short-term synaptic plasticity. *Proc. Natl. Acad. Sci. U.S.A.* 97 13372–13377. 10.1073/PNAS.230362997 11069288 PMC27231

[B9] Campbell S. L. Robel S. Cuddapah V. A. Robert S. Buckingham S. C. Kahle K. T. (2015). GABAergic disinhibition and impaired KCC2 cotransporter activity underlie tumor-associated epilepsy. *Glia* 63 23–36. 10.1002/GLIA.22730 25066727 PMC4237714

[B10] Chen S. Pickard J. D. Harris N. G. (2003). Time course of cellular pathology after controlled cortical impact injury. *Exp. Neurol.* 182 87–102. 10.1016/S0014-4886(03)00002-5 12821379

[B11] Cifu D. Hurley R. Peterson M. Cornis-Pop M. Rikli P. A. Ruff R. L. (2009). Clinical practice guideline: management of concussion/mild traumatic brain injury. *J. Rehabil. Res. Dev.* 46:C1. 10.1682/JRRD.2009.06.0076 20108447

[B12] Cooper G. M. (2000). “Protein degradation,” in *The Cell: A Molecular Approach*, 2nd Edn. ed. Oxford University Press (Sunderland, MA: Sinauer Associates). 10.1002/9781118539385.ch17

[B13] Cooper G. M. Coe B. P. Girirajan S. Rosenfeld J. A. Vu T. H. Baker C. (2011). A copy number variation morbidity map of developmental delay. *Nat. Genet.* 43 838–846. 10.1038/ng.909 21841781 PMC3171215

[B14] Dewan M. C. Rattani A. Gupta S. Baticulon R. E. Hung Y. C. Punchak M. (2018). Estimating the global incidence of traumatic brain injury. *J. Neurosurg.* 130 1080–1097. 10.3171/2017.10.JNS17352 29701556

[B15] Dittmeier M. Wassmuth K. Schuhmann M. K. Kraft P. Kleinschnitz C. Fluri F. (2016). Dabigatran etexilate reduces thrombin-induced inflammation and thrombus formation in experimental ischemic stroke. *Curr. Neurovasc. Res.* 13 199–206. 10.2174/1567202613666160517122605 27184031

[B16] Doherty C. P. O’Keefe E. Wallace E. Loftus T. Keaney J. Kealy J. (2016). Blood–brain barrier dysfunction as a hallmark pathology in chronic traumatic encephalopathy. *J. Neuropathol. Exp. Neurol.* 75:656. 10.1093/JNEN/NLW036 27245243 PMC4913433

[B17] Dunn-Meynell A. A. Levin B. E. (1997). Histological markers of neuronal, axonal and astrocytic changes after lateral rigid impact traumatic brain injury. *Brain Res.* 761 25–41. 10.1016/S0006-8993(97)00210-2 9247063

[B18] Ebrahimi S. Okabe S. (2014). Structural dynamics of dendritic spines: molecular composition, geometry and functional regulation. *Biochim. Biophys. Acta* 1838 2391–2398. 10.1016/J.BBAMEM.2014.06.002 24915021

[B19] Elgersma Y. Sweatt J. D. Giese K. P. (2004). Mouse genetic approaches to investigating calcium/calmodulin-dependent protein kinase ii function in plasticity and cognition. *J. Neurosci.* 24 8410–8415. 10.1523/JNEUROSCI.3622-04.2004 15456813 PMC6729904

[B20] Festoff B. W. Sajja R. K. van Dreden P. Cucullo L. (2016). HMGB1 and thrombin mediate the blood-brain barrier dysfunction acting as biomarkers of neuroinflammation and progression to neurodegeneration in Alzheimer’s disease. *J. Neuroinflammation* 13:194. 10.1186/S12974-016-0670-Z 27553758 PMC4995775

[B21] Filice F. Vörckel K. J. Sungur A. Ö Wöhr M. Schwaller B. (2016). Reduction in parvalbumin expression not loss of the parvalbumin-expressing GABA interneuron subpopulation in genetic parvalbumin and shank mouse models of autism. *Mol. Brain* 9:10. 10.1186/S13041-016-0192-8/METRICS 26819149 PMC4729132

[B22] Fornasiero E. F. Mandad S. Wildhagen H. Alevra M. Rammner B. Keihani S. (2018). Precisely measured protein lifetimes in the mouse brain reveal differences across tissues and subcellular fractions. *Nat. Commun.* 9:4230. 10.1038/s41467-018-06519-0 30315172 PMC6185916

[B23] Gainetdinov R. R. Hoener M. C. Berry M. D. (2018). Trace amines and their receptors. *Pharmacol. Rev.* 70 549–620. 10.1124/PR.117.015305 29941461

[B24] George K. K. Heithoff B. P. Shandra O. Robel S. (2021). Mild traumatic brain injury/concussion initiates an atypical astrocyte response caused by blood-brain barrier dysfunction. *J. Neurotrauma* 39 211–226. 10.1089/NEU.2021.0204 34806422 PMC8785769

[B25] Griffin A. D. Turtzo L. C. Parikh G. Y. Tolpygo A. Lodato Z. Moses A. D. (2019). Traumatic microbleeds suggest vascular injury and predict disability in traumatic brain injury. *Brain* 142 3550–3564. 10.1093/BRAIN/AWZ290 31608359 PMC6821371

[B26] Hayashi Y. Majewska A. K. (2005). Dendritic spine geometry: functional implication and regulation. *Neuron* 46 529–532. 10.1016/J.NEURON.2005.05.006 15944122

[B27] Hernandez M. L. Chatlos T. Gorse K. M. Lafrenaye A. D. (2019). Neuronal membrane disruption occurs late following diffuse brain trauma in rats and involves a subpopulation of NeuN negative cortical neurons. *Front. Neurol.* 10:1238. 10.3389/FNEUR.2019.01238/BIBTEX 31824411 PMC6883004

[B28] Ippolito D. M. Eroglu C. (2010). Quantifying synapses: an immunocytochemistry-based assay to quantify synapse number. *J. Visual. Exp.* 45:e2270. 10.3791/2270 21113117 PMC3159596

[B29] Irvine E. E. von Hertzen L. S. J. Plattner F. Giese K. P. (2006). A CaMKII autophosphorylation: a fast track to memory. *Trends Neurosci.* 29 459–465. 10.1016/J.TINS.2006.06.009 16806507

[B30] Ivens S. Kaufer D. Flores L. P. Bechmann I. Zumsteg D. Tomkins O. (2007). TGF-β receptor-mediated albumin uptake into astrocytes is involved in neocortical epileptogenesis. *Brain* 130 535–547. 10.1093/BRAIN/AWL317 17121744

[B31] Jha R. M. Kochanek P. M. Simard J. M. (2019). Pathophysiology and treatment of cerebral edema in traumatic brain injury. *Neuropharmacology* 145(Pt B):230. 10.1016/J.NEUROPHARM.2018.08.004 30086289 PMC6309515

[B32] Kim K. K. Adelstein R. S. Kawamoto S. (2009). Identification of neuronal nuclei (NeuN) as Fox-3, a new member of the fox-1 gene family of splicing factors. *J. Biol. Chem.* 284 31052–31061. 10.1074/JBC.M109.052969/ATTACHMENT/4C117D6B-1FE8-425B-8AF4-37A4876859B6/MMC1.PDF 19713214 PMC2781505

[B33] Lal D. Reinthaler E. M. Altmüller J. Toliat M. R. Thiele H. Nürnberg P. (2013). RBFOX1 and RBFOX3 mutations in rolandic epilepsy. *PLoS One* 8:e73323. 10.1371/JOURNAL.PONE.0073323 24039908 PMC3765197

[B34] Lin Y. S. Wang H. Y. Huang D. F. Hsieh P. F. Lin M. Y. Chou C. H. (2016). Neuronal splicing regulator RBFOX3 (NeuN) regulates adult hippocampal neurogenesis and synaptogenesis. *PLoS One* 11:e0164164. 10.1371/JOURNAL.PONE.0164164 27701470 PMC5049801

[B35] Liu W. Xu G. Z. Jiang C. H. Tian J. (2011). Macrophage colony-stimulating factor and its receptor signaling augment glycated albumin-induced retinal microglial inflammation in Vitro. *BMC Cell Biol.* 12:125. 10.1186/1471-2121-12-5/FIGURES/9 PMC3038972 21266043

[B36] Lucas C. H. Calvez M. Babu R. Brown A. (2014). Altered subcellular localization of the NeuN/Rbfox3 RNA splicing factor in HIV-Associated Neurocognitive Disorders (HAND). *Neurosci. Lett.* 558 97–102. 10.1016/J.NEULET.2013.10.037 24215932 PMC3880598

[B37] Lucchesi W. Mizuno K. Giese K. P. (2011). Novel insights into CaMKII function and regulation during memory formation. *Brain Res. Bull.* 85 2–8. 10.1016/J.BRAINRESBULL.2010.10.009 21070840

[B38] Marmarou A. Abd-Elfattah Foda M. A. Van den Brink W. Campbell J. Kita H. Demetriadou K. (1994). A new model of diffuse brain injury in rats. Part I: pathophysiology and biomechanics. *J. Neurosurg.* 80 291–300. 10.3171/jns.1994.80.2.0291 8283269

[B39] McPhail L. T. McBride C. B. McGraw J. Steeves J. D. Tetzlaff W. (2004). Axotomy abolishes NeuN expression in facial but not rubrospinal neurons. *Exp. Neurol.* 185 182–190. 10.1016/J.EXPNEUROL.2003.10.001 14697329

[B40] Merlini M. Rafalski V. A. Coronado P. E. R. Gill T. M. Ellisman M. Muthukumar G. (2019). Fibrinogen induces microglia-mediated spine elimination and cognitive impairment in an Alzheimer’s disease model. *Neuron* 101 1099–1108.e6. 10.1016/j.neuron.2019.01.014 30737131 PMC6602536

[B41] Mizee M. R. Wooldrik D. Lakeman K. A. M. van het Hof B. Drexhage J. A. R. Geerts D. (2013). Retinoic acid induces blood–brain barrier development. *J. Neurosci.* 33 1660–1671. 10.1523/JNEUROSCI.1338-12.2013 23345238 PMC6618717

[B42] Nichols J. N. Deshane A. S. Niedzielko T. L. Smith C. D. Floyd C. L. (2016). Greater neurobehavioral deficits occur in adult mice after repeated, as compared to single, mild traumatic brain injury (MTBI). *Behav. Brain Res.* 298 111–124. 10.1016/j.bbr.2015.10.052 26542813 PMC11817748

[B43] O’Keeffe E. Kelly E. Liu Y. Giordano C. Wallace E. Hynes M. (2020). Dynamic blood-brain barrier regulation in MILD TRAUMATIC BRAIN INJURY. *J. Neurotrauma* 37 347–356. 10.1089/neu.2019.6483 31702476 PMC10331162

[B44] Petersen M. A. Ryu J. K. Akassoglou K. (2018). Fibrinogen in neurological diseases: mechanisms, imaging and therapeutics. *Nat. Rev. Neurosci.* 19 283–301. 10.1038/NRN.2018.13 29618808 PMC6743980

[B45] Risher W. C. Ustunkaya T. Alvarado J. S. Eroglu C. (2014). Rapid golgi analysis method for efficient and unbiased classification of dendritic spines. *PLoS One* 9:107591. 10.1371/JOURNAL.PONE.0107591 25208214 PMC4160288

[B46] Robel S. Bardehle S. Lepier A. Brakebusch C. Götz M. (2011). Genetic deletion of Cdc42 reveals a crucial role for astrocyte recruitment to the injury site in vitro and in vivo. *J. Neurosci.* 31 12471–12482. 10.1523/JNEUROSCI.2696-11.2011 21880909 PMC6703275

[B47] Schachtrup C. Ryu J. K. Helmrick M. J. Vagena E. Galanakis D. K. Degen J. L. (2010). Fibrinogen triggers astrocyte scar formation by promoting the availability of active TGF-β after vascular damage. *J. Neurosci.* 30 5843–5854. 10.1523/JNEUROSCI.0137-10.2010 20427645 PMC2871011

[B48] Shandra O. Winemiller A. R. Heithoff B. P. Munoz-Ballester C. George K. K. Benko M. J. (2019). Repetitive diffuse mild traumatic brain injury causes an atypical astrocyte response and spontaneous recurrent seizures. *J. Neurosci.* 39 1944–1963. 10.1523/JNEUROSCI.1067-18.2018 30665946 PMC6407295

[B49] Tomkins O. Feintuch A. Benifla M. Cohen A. Friedman A. Shelef I. (2011). Blood-brain barrier breakdown following traumatic brain injury: a possible role in posttraumatic epilepsy. *Cardiovasc. Psychiatry Neurol*. 2011:765923. 10.1155/2011/765923 21436875 PMC3056210

[B50] Turtzo L. C. Luby M. Jikaria N. Griffin A. D. Greenman D. Bokkers R. P. H. (2021). Cytotoxic edema associated with hemorrhage predicts poor outcome after traumatic brain injury. *J. Neurotrauma* 38 3107–3118. 10.1089/NEU.2021.0037 34541886 PMC8820290

[B51] Ünal-Çevik I. Kilinç M. Gürsoy-Özdemir Y. Gurer G. Dalkara T. (2004). Loss of NeuN immunoreactivity after cerebral ischemia does not indicate neuronal cell loss: a cautionary note. *Brain Res.* 1015 169–174. 10.1016/J.BRAINRES.2004.04.032 15223381

[B52] Utami K. H. Hillmer A. M. Aksoy I. Chew E. G. Y. Teo A. S. M. Zhang Z. (2014). Detection of chromosomal breakpoints in patients with developmental delay and speech disorders. *PLoS One* 9:e90852. 10.1371/JOURNAL.PONE.0090852 24603971 PMC3946304

[B53] van Asch C. J. Luitse M. J. Rinkel G. J. van der Tweel I. Algra A. Klijn C. J. (2010). Incidence, case fatality, and functional outcome of intracerebral haemorrhage over time, according to age, sex, and ethnic origin: a systematic review and meta-analysis. *Lancet Neurol.* 9 167–176. 10.1016/S1474-4422(09)70340-0 20056489

[B54] Vigil F. A. Mizuno K. Lucchesi W. Valls-Comamala V. Giese K. P. (2017). Prevention of long-term memory loss after retrieval by an endogenous CaMKII inhibitor. *Sci. Rep.* 7:4040. 10.1038/S41598-017-04355-8 28642476 PMC5481336

[B55] Wang H. Y. Hsieh P. F. Huang D. F. Chin P. S. Chou C. H. Tung C. C. (2015). RBFOX3/NeuN Is required for hippocampal circuit balance and function. *Sci. Rep.* 5:17383. 10.1038/srep17383 26619789 PMC4664964

[B56] Witte M. E. Schumacher A. M. Mahler C. F. Bewersdorf J. P. Lehmitz J. Scheiter A. (2019). Calcium influx through plasma-membrane nanoruptures drives axon degeneration in a model of multiple sclerosis. *Neuron* 101 615–624.e5. 10.1016/J.NEURON.2018.12.023 30686733 PMC6389591

[B57] Wöhr M. Orduz D. Gregory P. Moreno H. Khan U. Vörckel K. J. (2015). Lack of parvalbumin in mice leads to behavioral deficits relevant to all human autism core symptoms and related neural morphofunctional abnormalities. *Transl. Psychiatry* 5:e525. 10.1038/tp.2015.19 25756808 PMC4354349

[B58] Wu Y. Wu H. Guo X. Pluimer B. Zhao Z. (2020). Blood–brain barrier dysfunction in mild traumatic brain injury: evidence from preclinical murine models. *Front. Physiol*. 11:1030. 10.3389/FPHYS.2020.01030 32973558 PMC7472692

[B59] Yoon S. Piguel N. H. Khalatyan N. Dionisio L. E. Savas J. N. Penzes P. (2021). Homer1 promotes dendritic spine growth through ankyrin-G and its loss reshapes the synaptic proteome. *Mol. Psychiatry* 26 1775–1789. 10.1038/s41380-020-00991-1 33398084 PMC8254828

[B60] Yuste R. Denk W. (1995). Dendritic spines as basic functional units of neuronal integration. *Nature* 375 682–684. 10.1038/375682a0 7791901

