# Supplementary material for: Mild Traumatic Brain Injury-Induced Disruption of the Blood-Brain Barrier Triggers an Atypical Neuronal Response
Source: Front Cell Neurosci. 2022 Feb 18;16:821885. doi: 10.3389/fncel.2022.821885 (PMC8894613; doi:10.3389/fncel.2022.821885)
Supplement: Supplementary file 1 [file Table_1.DOCX]

**Supplementary Table 1. Sex and genotype of mice used in experiments**

| **Figure** | **Animal #** | **Sex** | **Experimental Group** | **Timepoint** | **# of slices** | **Strain** |
| --- | --- | --- | --- | --- | --- | --- |
| **1** | **B0641** | **M** | **1x TBI** | **Instant** | **3** | **C57/Bl6** |
| **1** | **B0603** | **M** | **1x TBI** | **Instant** | **4** | **C57/Bl6** |
| **1** | **B0752** | **F** | **1x TBi** | **Instant** | **3** | **C57/Bl6** |
| **1** | **B0721** | **F** | **1x TBI** | **Instant** | **3** | **C57/Bl6** |
| **1** | **B0722** | **F** | **1x TBI** | **Instant** | **5** | **C57/Bl6** |
| **1** | **5558** | **M** | **3x TBI** | **7 dpi** | **3** | **C57/Bl6** |
| **1** | **5563** | **M** | **3x TBI** | **7 dpi** | **4** | **C57/Bl6** |
| **1** | **B0742** | **F** | **3x TBI** | **7 dpi** | **3** | **C57/Bl6** |
| **1** | **B0869** | **F** | **3x TBI** | **7 dpi** | **4** | **C57/Bl6** |
| **1** | **B0739** | **F** | **3x TBI** | **7 dpi** | **4** | **C57/Bl6** |
| **1** | **B0740** | **F** | **3x TBI** | **7 dpi** | **3** | **C57/Bl6** |
| **1** | **5574** | **F** | **3x TBI** | **7 dpi** | **4** | **C57/Bl6** |
| **1** | **5566** | **M** | **naive** | **7 dpi** | **7** | **C57/Bl6** |
| **1** | **5567** | **F** | **naive** | **7 dpi** | **7** | **C57/Bl6** |
| **1** | **B0642** | **M** | **Sham** | **Instant** | **8** | **C57/Bl6** |
| **1** | **B0723** | **F** | **Sham** | **Instant** | **8** | **C57/Bl6** |
| **1** | **B0868** | **F** | **Sham** | **7 dpi** | **7** | **C57/Bl6** |
| **1** | **B0870** | **F** | **Sham** | **7dpi** | **6** | **C57/Bl6** |
| **2** | **5557** | **M** | **3x TBI** | **7 dpi** | **7** | **C57/Bl6** |
| **2** | **5556** | **M** | **3x TBI** | **7 dpi** | **4** | **C57/Bl6** |
| **2** | **B0739** | **F** | **3x TBI** | **7 dpi** | **3** | **C57/Bl6** |
| **2** | **B0740** | **F** | **3x TBI** | **7 dpi** | **3** | **C57/Bl6** |
| **2** | **B0869** | **F** | **3x TBI** | **7 dpi** | **3** | **C57/Bl6** |
| **2** | **B0742** | **F** | **3x TBI** | **7 dpi** | **3** | **C57/Bl6** |
| **2** | **B0603** | **M** | **1x TBI** | **Instant** | **3** | **C57/Bl6** |
| **2** | **B0721** | **F** | **1x TBI** | **Instant** | **5** | **C57/Bl6** |
| **2** | **B0722** | **F** | **1x TBI** | **Instant** | **3** | **C57/Bl6** |
| **2** | **B0752** | **F** | **1x TBI** | **Instant** | **3** | **C57/Bl6** |
| **2** | **B0724** | **F** | **1x TBI** | **Instant** | **3** | **C57/Bl6** |
| **2** | **5566** | **M** | **naive** | **7 dpi** | **7** | **C57/Bl6** |
| **2** | **5567** | **F** | **naive** | **7 dpi** | **6** | **C57/Bl6** |
| **2** | **B0642** | **M** | **Sham** | **Instant** | **11** | **C57/Bl6** |
| **2** | **B0725** | **M** | **Sham** | **Instant** | **8** | **C57/Bl6** |
| **2** | **B0868** | **F** | **Sham** | **7 dpi** | **7** | **C57/Bl6** |
| **3** | **B0752** | **F** | **1x TBI** | **Instant** | **3** | **C57Bl/6** |
| **3** | **B0960** | **F** | **1x TBI** | **Instant** | **3** | **C57Bl/6** |
| **3** | **B0721** | **F** | **1x TBI** | **Instant** | **3** | **C57Bl/6** |
| **3** | **B0724** | **F** | **1x TBI** | **Instant** | **1** | **C57Bl/6** |
| **3** | **B0739** | **F** | **3x TBI** | **7 dpi** | **3** | [**C57Bl/6**](https://my.labguru.com/biocollections/rodent_strains/81) |
| **3** | **B0740** | **F** | **3x TBI** | **7 dpi** | **3** | **C57Bl/6** |
| **3** | **B0742** | **F** | **3x TBI** | **7 dpi** | **4** | **C57Bl/6** |
| **3** | **B0686** | **M** | **SHAM** | **Instant** | **4** | **C57Bl/6** |
| **3** | **B0919** | **M** | **SHAM** | **Instant** | **6** | **C57Bl/6** |
| **3** | **B0925** | **F** | **SHAM** | **Instant** | **7** | **C57Bl/6** |
| **3** | **B0870** | **F** | **SHAM** | **7 dpi** | **6** | **C57Bl/6** |
| **3** | **B1172** | **F** | **SHAM** | **7 dpi** | **5** | **C57Bl/6** |
| **3** | **B1117** | **F** | **3x TBI** | **7 dpi** | **3** | **C57Bl/6** |
| **3** | **B1174** | **F** | **3x TBI** | **7 dpi** | **4** | **C57Bl/6** |
| **3** | **B0923** | **F** | **1x TBI** | **Instant** | **4** | **C57Bl/6** |
| **4** | **B0676** | **M** | **3x TBI** | **7 dpi** | **4** | **C57Bl/6** |
| **4** | **B0677** | **M** | **3x TBI** | **7 dpi** | **7** | **C57Bl/6** |
| **4** | **B1230** | **M** | **3x TBI** | **7 dpi** | **8** | **C57Bl/6** |
| **4** | **B0680** | **M** | **Sham** | **7 dpi** | **9** | **C57Bl/6** |
| **4** | **B0681** | **M** | **Sham** | **7 dpi** | **1** | **C57Bl/6** |
| **4** | **B0736** | **M** | **Sham** | **7 dpi** | **5** | **C57Bl/6** |
| **5** | **A9453** | **M** | **TX 1** | **control** | **7** | **DTA-flx//Cdh5(PAC)-cre** |
| **5** | **A9456** | **F** | **TX 1** | **control** | **6** | **DTA-flx//Cdh5(PAC)-cre** |
| **5** | **B0877** | **F** | **TX 1** | **control** | **7** | **DTA-flx//Cdh5(PAC)-cre** |
| **5** | **B0878** | **M** | **TX 1** | **6 hpa** | **7** | **DTA-flx//Cdh5(PAC)-cre** |
| **5** | **B0876** | **F** | **TX 1** | **6 hpa** | **7** | **DTA-flx//Cdh5(PAC)-cre** |
| **6** | **2310** | **M** | **3x TBI** | **6 mopi** | **3** | **C57Bl/6** |
| **6** | **2311** | **M** | **3x TBI** | **6 mopi** | **3** | **C57Bl/6** |
| **6** | **2314** | **M** | **3x TBI** | **6 mopi** | **3** | **C57Bl/6** |
| **6** | **2302** | **F** | **3x TBI** | **6 mopi** | **4** | **C57Bl/6** |
| **6** | **2312** | **M** | **SHAM** | **6 mopi** | **3** | **C57Bl/6** |
| **6** | **2313** | **M** | **SHAM** | **6 mopi** | **3** | **C57Bl/6** |
| **6** | **2301** | **F** | **SHAM** | **6 mopi** | **4** | **C57Bl/6** |
| **6** | **2303** | **F** | **SHAM** | **6 mopi** | **4** | **C57Bl/6** |
